# Chromosome-Scale Assembly of the Complete Genome Sequence of *Leishmania* (*Mundinia*) sp. Ghana, Isolate GH5, Strain LV757

**DOI:** 10.1128/MRA.00591-21

**Published:** 2021-09-30

**Authors:** Hatim Almutairi, Michael D. Urbaniak, Michelle D. Bates, Godwin Kwakye-Nuako, Waleed S. Al-Salem, Rod J. Dillon, Paul A. Bates, Derek Gatherer

**Affiliations:** a Division of Biomedical & Life Sciences, Faculty of Health & Medicine, Lancaster University, Lancaster, United Kingdom; b Ministry of Health, Riyadh, Saudi Arabia; c Department of Biomedical Sciences, School of Allied Health Sciences, College of Health & Allied Sciences, University of Cape Coast, Cape Coast, Ghana; University of California, Riverside

## Abstract

*Leishmania* (*Mundinia*) sp. Ghana is a kinetoplastid parasite isolated in 2015 in Ghana. We report the complete genome sequence of *L.* (*M.*) sp. Ghana, sequenced using combined short-read and long-read technologies. This will facilitate greater understanding of this novel pathogen and its relationships within the subgenus *Mundinia*.

## ANNOUNCEMENT

In 2015, a hitherto unknown parasite of the genus *Leishmania* was detected in a case of human cutaneous leishmaniasis in Ghana (1). This putative new species has not yet been formally named but was classified as the fifth species in the recently established subgenus *Mundinia* (2, 3), which also includes Leishmania enriettii (2), Leishmania macropodum (4), Leishmania orientalis (5), and Leishmania martiniquensis (6). Phylogenetic analyses indicate that the subgenus *Mundinia* is the sister group to the other *Leishmania* subgenera (1, 7). Furthermore, *Mundinia* species are found on every continent except Antarctica (8), supporting the hypothesis of evolution from a common ancestor prior to the division of the Gondwana supercontinent (9). We report the complete genome assembly and annotation of *L.* (*M.*) sp. Ghana, isolate GH5, strain LV757 (WHO code MHOM/GH/2012/GH5;LV757). This will contribute to research on the origins and expansion of *Mundinia*.

Parasites were grown using an *in vitro* culture system previously developed for *L.* (*M.*) *orientalis* axenic amastigotes (10) in Schneider’s insect medium at 26°C as promastigotes, then in M199 medium supplemented with 10% fetal calf serum (FCS), 2% stable human urine, 1% basal medium Eagle vitamins, and 25 μg/ml gentamicin sulfate, with subpassage to fresh medium every 4 days to sustain parasite growth and viability. DNA was extracted and purified using a Qiagen DNeasy blood and tissue kit using the spin column protocol, according to the manufacturer’s instructions. The extracted DNA concentration was assessed using a Qubit fluorometer, microplate reader, and agarose gel electrophoresis. All sequencing libraries were based on the same extracted DNA sample to avoid any inconsistency.

Short-read library construction and sequencing were contracted to (i) BGI (Shenzhen, China), who constructed DNBSEQ libraries producing paired-end reads (270 bp and 500 bp) using the Illumina HiSeq platform, and (ii) Aberystwyth University (Aberystwyth, UK), who constructed TruSeq Nano DNA libraries producing paired-end reads (300 bp) using the Illumina MiSeq platform. We performed long-read library preparation and sequencing according to the Nanopore protocol (SQK-LSK109) on R9 flow cells (FLO-MIN106). The read quality was assessed using MultiQC (11), incorporating the use of FastQC for the Illumina short reads and pycoQC for the Nanopore long reads.

We assembled the long reads using Flye (12), with default parameters, to generate chromosome-scale scaffolds. Then, using Minimap2 (13) and SAMtools (14), we mapped the short reads onto the assembled scaffolds to compensate for erroneous bases within the long reads and create consensus sequences. After polishing the assembly using Pilon (15), another round of consensus short-read mapping was performed. Then, we removed duplicated contigs and sorted the remainder according to length using Funannotate (16). Finally, we separated the chimeric sequences and performed scaffolding using RaGOO (17), with the L. major Friedlin strain genome (GenBank accession number GCA_000002725.2) (18) as a reference guide, aligning all 36 chromosomes for our assembly, thereby also determining the chromosome ends to be complete, with the exception of 80 unplaced contigs totaling 1,077,537 bp.

**FIG 1:**
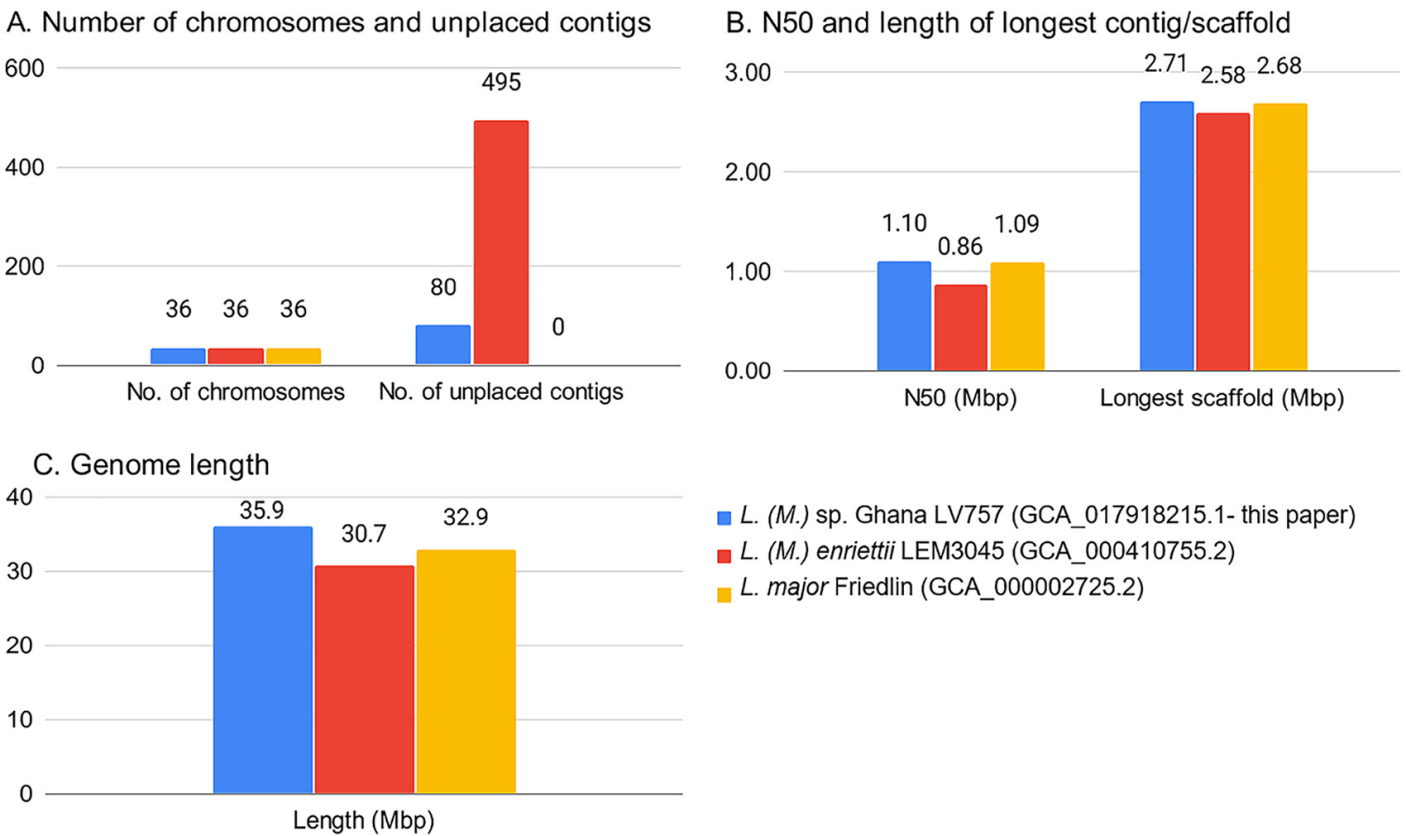
Assembly comparison of *L.* (*M.*) sp. Ghana LV757 with *L.* (*M.*) *enriettii* LEM3045 and L. major Friedlin.

The analysis workflow for assembly and annotation was performed using Snakemake (19) and is available online for reproducibility purposes (https://github.com/hatimalmutairi/LGAAP), including the software versions and parameters used (20). Figure 1 compares our assembly with other complete genome sequences.

We assessed the assembly completeness using BUSCO (21), with the lineage data set for the phylum *Euglenozoa*, containing 130 single-copy orthologs from 31 species, and found 123 of these to be present (94.6% completeness). We carried out functional annotation and prediction using the MAKER2 (22) annotation pipeline in combination with AUGUSTUS (23) gene prediction software. Table 1 shows further summary metrics for the sequencing, assembly, and annotation.

**TABLE 1:** Detailed summary metrics of the genome sequencing, assembly, and annotation for *L.* (*M.*) sp. Ghana LV757

Feature(s)	Metric(s)
Total no. of reads	49,308,106
No. of MiSeq reads	5,195,324
No. of HiSeq reads	43,244,422
MinION reads (bp)	868,360
MinION read *N*_50_ (bp)	19,170
Bases (Gb)	26.93
Genome coverage (×)	371.2
Total no. of scaffolds	116
Genome size (bp)	35,953,538
*N*_50_ (bp)	1,100,365
% GC content	59.70
No. of Ns (% of genome)	481 (0.001)
No. of genes	8,119
Gene density (Mb)	225.8
No. of exons	8,119
Mean gene length (bp)	1,838
Total length of CDSs^*a*^ (Mb) (% of genome)	14.92 (41.51)

a CDSs, coding DNA sequences.

### Data availability.

The assembly and annotations are available under GenBank assembly accession number GCA_017918215.1. The master record for the whole-genome sequencing project is available under accession number JAFJZN000000000.1. The raw sequence reads are available under BioProject accession number PRJNA691536.
